# Adaptations for Pressure and Temperature in Dihydrofolate Reductases

**DOI:** 10.3390/microorganisms9081706

**Published:** 2021-08-11

**Authors:** Ryan W. Penhallurick, Maya D. Durnal, Alliyah Harold, Toshiko Ichiye

**Affiliations:** Department of Chemistry, Georgetown University, Washington, DC 20057, USA; rwp33@georgetown.edu (R.W.P.); mdd96@georgetown.edu (M.D.D.); ah1408@georgetown.edu (A.H.)

**Keywords:** molecular dynamics simulations, extremophiles, piezophiles, hydrogen bonds

## Abstract

Enzymes from extremophilic microbes that live in extreme conditions are generally adapted so that they function under those conditions, although adaptations for extreme temperatures and pressures can be difficult to unravel. Previous studies have shown mutation of Asp27 in *Escherichia coli* dihydrofolate reductase (DHFR) to Glu27 in *Moritella profunda* (Mp). DHFR enhances activity at higher pressures, although this may be an adaptation for cold. Interestingly, MpDHFR unfolds at ~70 MPa, while *Moritella yayanosii* (My) was isolated at depths corresponding to ~110 MPa, indicating that MyDHFR might be adapted for higher pressures. Here, these adaptations are examined using molecular dynamics simulations of DHFR from different microbes in the context of not only experimental studies of activity and stability of the protein but also the evolutionary history of the microbe. Results suggest Tyr103 of MyDHFR may be an adaptation for high pressure since Cys103 in helix F of MpDHFR forms an intra-helix hydrogen bond with Ile99 while Tyr103 in helix F of MyDHFR forms a hydrogen bond with Leu78 in helix E. This suggests the hydrogen bond between helices F and E in MyDHFR might prevent distortion at higher pressures.

## 1. Introduction

The discoveries of “extremophilic” organisms that thrive under extremes of temperature, pressure, and other conditions [1] raise questions about the nature of adaptations in their biomolecules so that they can function under conditions where their counterparts from mesophiles would fail. Determining the adaptations of proteins for extreme conditions can lead to a greater fundamental understanding of structure-function relationships in proteins, as well as practical applications such as bioengineering proteins to function under specific conditions [2]. In addition, determining the limiting conditions where enzyme activity can be maintained may be useful in defining conditions for the “limits of life” to guide the search for life in extreme environments such as beneath the oceanic and continental surface or even extraterrestrially.

Studies have often focused on how enzymes from extremophiles function under extreme conditions since enzyme activity is a requirement for growth. Perhaps the best understood extremes are temperatures. Psychrophilic (cold-loving) microbes have been found growing at temperatures as low as −20 °C [3], while thermophilic (hot-loving) microbes can grow at temperatures as high as 122 °C [4]. Consistent with the hypothesis that enzyme activity is similar at “corresponding states” of their microbial source, namely, the growth temperature *T*_G_ of the microbe [5,6], homologous enzymes from psychrophiles, mesophiles, and thermophiles, often have maximum activity near the *T*_G_ of the microbes [7,8]. Enzymes from thermophiles apparently need more stabilizing interactions so that they do not unfold at the high *T*_G_ of their organism, while enzymes from psychrophiles often have fewer stabilizing interactions, which have been suggested to promote flexibility for activity at the low *T*_G_ of their organism. Thus, a balance between stability and flexibility might be necessary since more interactions promote stability while fewer interactions promote flexibility, giving rise to the maximum activity near *T*_G_ [7]. However, fewer interactions in psychrophiles might also represent a random loss without a driving force. For instance, comparison of a dihydrofolate reductase (DHFR) from a mesophile with that from a thermophile shows greater flexibility of the thermophile DHFR [9]. Interestingly, fewer interactions also imply less stability to both heat- and cold-unfolding so that proteins from psychrophiles may have cold-unfolding temperatures that are actually higher than those of proteins from mesophiles or thermophiles. Thus, selection against cold-unfolding does not generally appear to be a driving force for psychrophilic proteins as long as the cold-unfolding temperature is lower than the organism’s *T*_G_. Other modes of adapting to cold temperatures include lowering activation energy barriers [10,11,12].

Understanding adaptation to extreme conditions should also consider whether the evolution was toward or away from the extreme [13]. These studies show that proteins from hyperthermophilic Archaea, which are thought to have originated in a hot environment, tend to have “structure-based” adaptation to high temperatures in that they tend to be compact due to more stabilizing interactions and more hydrophobic residues than proteins from mesophiles, which have, over time, experienced random mutations that eventually made them less suited for hot environments. On the other hand, proteins from mesophiles that evolved in a normal environment but later recolonized hot environments tend to have “sequence-based” adaptations involving a small number of strong interactions.

Of extreme conditions, the effects of high pressure have been relatively unexplored, because of the difficulties both in producing high pressure in the laboratory and in collecting samples of piezophiles (high-pressure loving). Thus, the development of high-pressure biophysical instrumentation [14,15,16,17,18,19,20] and greater sampling of microbes from the deepest ocean and far beneath the continental surface at pressures beyond 1 kbar [21] offer new opportunities to explore this variable. While growth is inhibited in many mesophilic microbes at about 400 to 500 bar [22], more recent studies indicate that microbes can grow in the 10 kbar range [19,23,24]. Some microbes from the cold deep-sea are obligate piezophiles that can live at pressures near 1 kbar but do not grow at 1 bar [25]. Interestingly, archaea have been found near hydrothermal vents at 1 bar that thrive at pressures up to 1.2 kbar [26].

For proteins, the effects of pressure are compression and denaturation [22]. At pressures below 4 kbar, compression dominates, while at pressures well above 4 kbar, denaturation can occur. One of the most experimentally studied enzymes for both structure and activity under pressure is the ubiquitous enzyme, DHFR, which reduces dihydrofolate (DHF) to tetrahydrofolate (THF). DHFR from *Escherichia coli* (Ec) is well-characterized experimentally under mesophilic conditions. Structures of EcDHFR at 1 bar bound with different combinations of the oxidized/reduced co-factor nicotinamide adenine dinucleotide phosphate (NADP^+^/NADPH), and DHF, THF, or analogs have contributed to a detailed picture of the molecular mechanism of DHFR [27]. For instance, conformational changes between “occluded”, “closed”, and “open” conformations of the Met20 loop at the nicotinamide binding site have been implicated in the catalytic activity [28,29]. Interestingly, although only the occluded conformation is seen in THF-bound DHFR by NMR at 1 bar, a high-pressure ^15^N/^1^H two-dimensional NMR study has demonstrated the appearance of a second conformation as pressure is increased up to 2 kbar [30]. In addition, crystal structures of EcDHFR at pressures up to 8 kbar [31] indicate that transient open conformations appear important for nicotinamide to enter its binding pocket.

**Figure 1:**
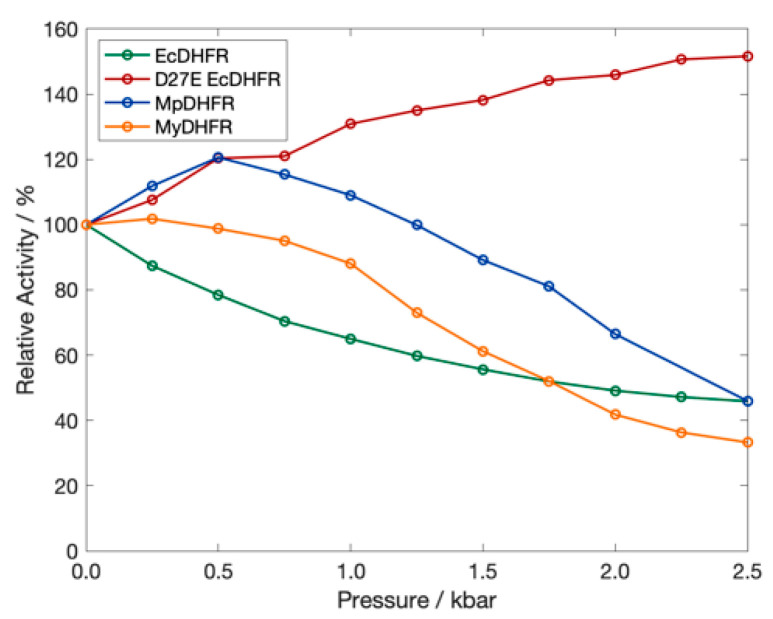
The relative activity of DHFR from *E. coli* (green), *M. profunda* (blue), *M. yayanosii* (orange) and of D27E EcDHFR (red) as a function of pressure at 298 K. Data from Refs. [32,35,36].

To understand adaptations for pressure, thorough experimental comparisons have also been made of the pressure dependence of the activity and stability of DHFR from a moderate piezophile and a mesophile [32]; specifically, from *Moritella profunda* (Mp), with an optimal growth pressure *P*_G_ of ~220 bar at 6 °C [33], and *E. coli*, with a presumed *T*_G_ of 37 °C at 1 bar. Although structural differences are not apparent between crystal structures of MpDHFR, EcDHFR [32] or other homologous proteins from piezophiles and mesophiles [34], wild-type MpDHFR has maximum enzyme activity at 500 bar while wild-type EcDHFR is monotonically inactivated by pressure above 1 bar (Figure 1).

One adaptation in MpDHFR that might appear to be for pressure is the presence of Glu27 rather than Asp27 in EcDHFR. With increasing pressure, the Asp27Glu mutation (D27E) of EcDHFR exhibits increased activity, rather than the decreased activity observed in wild-type EcDHFR (Figure 1) [36]. However, enzyme activity does not always increase with pressure for DHFR from other deep-sea bacteria [36]. Since many of the piezophile proteins studied have been from microbes from the cold deep ocean, the adaptations may be for cold temperature rather than for high pressure [37]. Recently, a combined experimental and simulation study comparing adenylate kinase (Adk) from *E. coli* and *Photobacterium profundum* SS9, a piezophile, also identified specific residue adaptations that make EcAdk more active under pressure [38]. However, since *P. profundum* is a deep-sea microbe with optimal *T*_G_ = 15 °C and *P*_G_ = 280 bar [39], it is also not clear if the adaptation was for high pressure or cold temperature.

**Figure 2:**
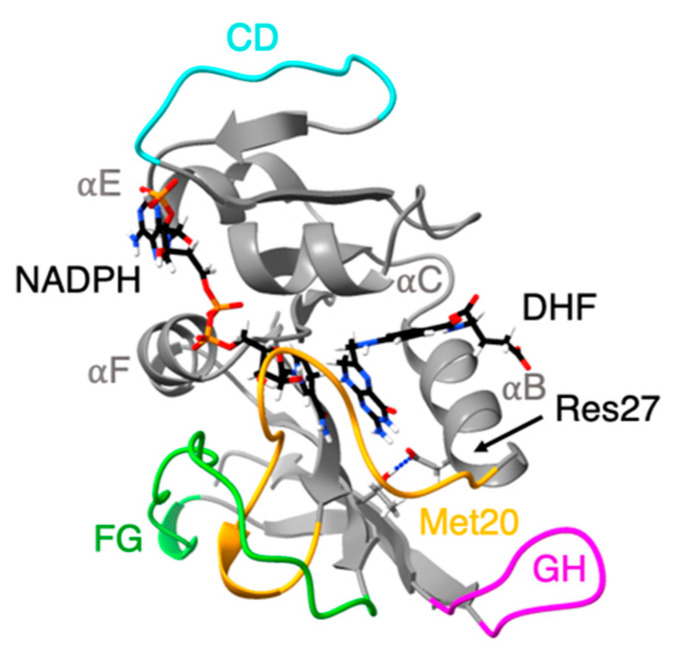
Ribbon representation of the structure of *E. coli* dihydrofolate reductase: Met20 (yellow), CD (blue), FG (green) and GH (pink) loops are highlighted. Cofactor (NADPH) and ligand (DHF) shown in stick representation. Thr113 O*_γ_*…Res27 hydrogen bond shown as dashed line.

We have also been examining DHFR from piezophiles compared to mesophiles, using molecular dynamics (MD) simulations. Our early comparisons were of pressure and temperature effects on MpDHFR and EcDHFR in MD simulations. These studies showed that the average root mean-square atomic fluctuations of either DHFR increased with temperature, were generally higher at 200 bar than 1 bar, and were greater for MpDHFR than EcDHFR at any given temperature and pressure [40]. A quasi-harmonic analysis of the underlying potential surface showed that the underlying potential surface had steeper wells in EcDHFR than MpDHFR [41]. At a given set of conditions, the average number of hydrogen bonds was consistently slightly higher in EcDHFR than MpDHFR (i.e., at 279 K and 1 bar, 107 for EcDHFR vs. 103 for MpDHFR) [42]. Intriguingly, a significant difference was that the Thr113…Asp27 hydrogen bond in EcDHFR had a long ~25 ns lifetime at 279 K while the equivalent Thr113…Glu27 hydrogen bond in MpDHFR had a short 87 ps lifetime at 279 K (Figure 2). Covariance matrices indicate that the strength of the 113-27 hydrogen bond may affect the activity via the correlation of the Met20 and GH loops [43].

Our recent simulations of wild-type and D27E EcDHFR [44] indicate that the correlations of the Met20 loop of wild-type EcDHFR at 1 bar are similar to D27E DHFR at 220 bar. Apparently, the Thr113 O*_γ_*…Asp27 O*_δ_* hydrogen bond is less flexible than a Thr113 O*_γ_*…Glu27 O*_ε_* hydrogen bond because of the extra carbon in glutamine side chain, but pressure increases correlation so that the Thr113 O*_γ_*…Asp27 O*_δ_* hydrogen bond of EcDHFR becomes too strong while the Thr113 O*_γ_*…Glu27 O*_ε_* hydrogen bond in the mutant becomes more like the Thr113 O*_γ_*…Asp27 O*_δ_* of EcDHFR at 1 bar. The flexibility of the Thr113-Res27 affects the correlation of the GH and Met20 loops, and NMR studies that indicate proper coupling between these two loops is necessary for proper functioning of DHFR [45].

**Figure 3:**
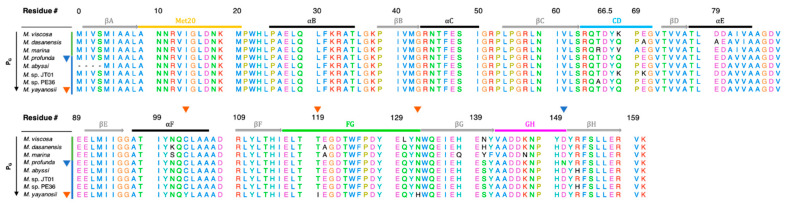
Sequence alignment of *Moritella* DHFRs sorted from lowest to highest *P*_G_. Unique residues are indicated by triangles for MpDHFR (blue), and MyDHFR (yellow). Secondary structure elements are indicated by horizontal bars: α-helices (gray), β-strands (black) and the Met20 (yellow), CD (blue), FG (green) and GH (magenta) loops. *M. japonica* DHFR left out due to low sequence identity (28.85%) to MpDHFR. Sequence numbering is based on *E. coli* DHFR, with the insertion in *Moritella* DHFRs between residues 65 and 66 of *E. coli* DHFR denoted as 66.5.

DHFR from the genus *Moritella* present an opportunity for studying sequence adaptations for piezophilicity since their sequences are highly homologous (Figure 3) and represent a wide range of depths from the surface to the deepest known point in the ocean, ~11 km into the Mariana Trench [25]. *M. yayanosii* (My), which has the deepest isolation depth corresponding to 1.1 kbar, is an obligate piezophile with an optimal *P*_G_ of 0.8 kbar at 10 °C [25] yet unbound (apo)-MpDHFR has been shown to unfold at pressures of ~0.7 kbar [32]. In addition, for molecular dynamics simulations, there is a crystal structure for MpDHFR and there are only four sequence differences between MpDHFR and MyDHFR so that a reliable homology model for MyDHFR is feasible.

Here, a general framework is presented to understand adaptations in enzymes from extremophiles in light of the evolutionary history of the microbes from which they come. New molecular dynamics simulations of MpDHFR and MyDHFR at 1 and 800 bar and at 279 K, and results from previous simulations of EcDHFR and D27E EcDHFR at 1 and 220 bar and at 279 K [44] are discussed in light of this framework as well as experimental studies of the proteins. The hydrogen bonding patterns in the simulations of MpDHFR and MyDHFR are compared, focusing on the four sequence differences between MpDHFR and MyDHFR (Figure 3); specifically, MpDHFR has Cys103, T119, N132, and N150, while MyDHFR has Tyr103, Ile119, H132, and D150. Since Tyr103, Ile119, and H132 of MyDHFR are unique among the other *Moritella* DHFR, one may be an adaptation for high pressure.

## 2. Methods

The calculations were performed similar to previous work [44] so are described only briefly here and more thoroughly in the Supplementary Information. All DHFR are bound to DHF and NADPH. Coordinate manipulations and analyses were performed using the molecular mechanics package CHARMM version 40b1 [46] using CHARMM-GUI [47] for the set up. Molecular dynamics (MD) simulations were performed using the molecular mechanics package OpenMM version 7.3.1 [48] compiled with CUDA version 9.2. Note that our earlier work [40,42,43] used different procedures including use of CHARMM for the molecular dynamics and DHFR was in association with tetrahydrofolate and no co-factor. The protein was modeled using the CHARMM36 all-atom non-polarizable potential energy parameter set [49,50]. Water was modeled by TIP4P-Ew [51] because of the importance of modeling changes in the properties of water under pressure. DHF was modeled using the CHARMM General Force Field (CGenFF) generated through ParamChem [52] as in our previous work [40] and NADPH was modeled as in other work [53].

BLASTp [54] was used to identify and score non-redundant DHFR protein sequences with respect to MpDHFR. Sequences were aligned using ClustalX v.2 [55]. Correction of the first residue, addition of the C-terminal tail and all mutations were built using GalaxyFill [56] in PDB Reader [57] of CHARMM-GUI. Coordinates for the MpDHFR, cofactor, folate and crystallographic waters used during homology modeling were obtained from the Protein Data Bank from the NADP^+^/folate-bound MpDHFR crystal structure (PDB ID: 2ZZA) [32]. The aligned sequences from MpDHFR and MyDHFR were used for the resulting models [58,59]. Ligand Reader and Modeler [60] in CHARMM-GUI was used to modify the pterin ring of folate from a planar system to the partially-puckered ring of dihydrofolate (DHF), as well as to modify NADP^+^ to NADPH. Coordinates for the proteins were generated with PDB Reader of CHARMM-GUI; specifically, termini were capped with amino and carboxyl groups, and missing hydrogen coordinates built. Crystal waters within 2.5 Å of any modeled residue were deleted.

The DHFRs were first solvated in a cubic simulation box of equilibrated TIP4P-Ew with a distance between faces of ~70 Å. The smallest distance from a protein atom to a side of the box was ~10 Å. The proteins were then neutralized in 0.15 M KCl using the Monte-Carlo placement method. The final systems are described in Table S1 of the Supplementary Materials.

The simulations were in “mixed precision” with a 0.001 ps time step. The Leonard-Jones interactions were switched off using the default OpenMM switching function from 10 Å to 12 Å and no long-range corrections. The particle mesh Ewald (PME) summation algorithm [61], with an Ewald error tolerance of 1 × 10^−5^, was used for the electrostatics. Prior to molecular dynamics, each system was minimized with 500 iterations of the L-BFGS algorithm with a harmonic restraint with a force constant of 100 kcal mol^−1^ Å^−2^ on the heavy atoms of the protein backbone. After heating and pressuring as in previous work [44], the system was equilibrated for another 5 ns followed by 50 ns of production run in the *NVT* ensemble.

## 3. Analysis

Average properties were calculated from coordinates written at 1 ps intervals. The root-mean-squared fluctuations of protein heavy atoms 〈Δ*r*_HA_^2^〉 were calculated with respect to the average structure within 5 ns blocks. Hydrogen bonds were defined as having a distance between the donor atom *i* and acceptor atom *j* smaller than 2.40 Å and the angle of D–H…A larger than 130°. The average number of hydrogen bonds, *N*_HB_, was the average over the simulation of the number of hydrogen bonds at each timestep. Two hydrogen bonds simultaneously formed with the same protein atom were calculated as two separate events. The statistics for the 5 ns 〈Δ*r*_HA_^2^〉 and number of hydrogen bonds were obtained from block averaging the 5 ns blocks.

## 4. Results

Overall, the structures of the wild-type and mutant EcDHFR and of the two *Moritella* DHFR are quite similar in the simulations. At 1 bar, the root mean-square deviation between EcDHFR and D27E EcDHFR is 0.35 Å and between MpDHFR and MyDHFR is 0.58 Å and is mainly due to slight differences in the conformations of the Met20, FG, and GH loops, which are known to be mobile. The average number of hydrogen bond and atomic fluctuations found in the simulations of DHFR (Table 1) are also quite similar. Generally, a greater number of hydrogen bonds is associated with smaller fluctuations, although, again, the error bars are large. More notably, when comparing hydrogen bonds with greater than 50% occupancy in the simulations at 1 bar, 87 appear to be common between EcDHFR and D27E EcDHFR, 84 are common between MpDHFR and MyDHFR, and most of these are common between all 4 DHFR. Of the ones that were not common in the EcDHFR, 7 had a hydrogen bond in EcDHFR but not in D27E EcDHFR while 4 had a hydrogen bond in D27E EcDHFR but not EcDHFR, consistent with Table 1, and of the ones that were not common between MpDHFR and MyDHFR, 4 had a hydrogen bond in MpDHFR and not in MyDHFR and 6 had hydrogen bonds in MyDHFR and not in MpDHFR, also consistent with Table 1. As a whole, a lesser number of hydrogen bonds seemed more indicative coming from a cooler environment than larger atomic fluctuations, except for in MyDHFR, which may reflect the need for protection against distortion. Thus, specific hydrogen bonds are examined next.

**Figure 4:**
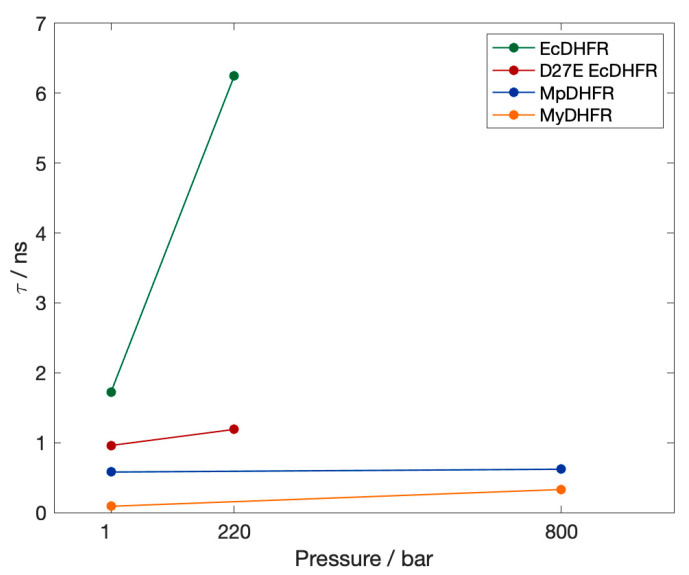
The lifetime *τ* of the hydrogen bond between Thr113 O*_γ_*…Res27 at 1 bar in the simulations at 279 K. The occupancy is greater than 0.98 in all cases.

First, the lifetime *τ* of the hydrogen bond between residues 113 and 27 in the simulations at 279 K (Figure 4) are compared. Since the occupancies are all close to 1, the lifetimes are an indication of the relative strength of the hydrogen bonds. Thr113 O*_γ_*…Asp27 O*_δ_* in EcDHFR is much stronger than Thr113 O*_γ_*…Glu27 O*_ε_* in D27E EcDHFR and either *Moritella* DHFR, with the Thr113…Res27 (Res being either Asp or Glu) strengthening with pressure regardless of the microbial source. Thus, as pressure is increased, Thr113 O*_γ_*…Asp27 O*_δ_* in EcDHFR becomes almost unbreakable while Thr113 O*_γ_*…Glu27 O*_ε_* in D27E EcDHFR and either *Moritella* DHFR becomes more like Thr113 O*_γ_*…Asp27 O*_δ_* in EcDHFR at 1 bar. Interestingly, the lifetime of Thr113 O*_γ_*…Glu27 O*_ε_* in D27E is shorter in MyDHFR than in MpDHFR, even though there are only 4 sequence differences between MyDHFR and MpDHFR.

**Figure 5:**
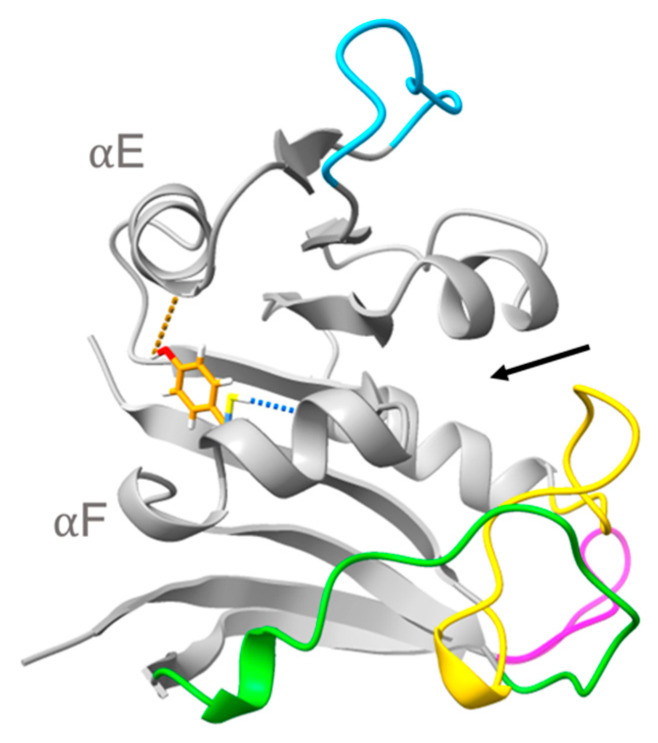
Superimposed structures of *M. profunda* and *M. yayanosii* dihydrofolate reductase highlighting Cys103 S*_γ_*…Ile99 O in MpDHFR (blue) and Tyr103 O*_η…_*Leu78 O in MyDHFR (orange) hydrogen bonds (denoted with dashed blue lines). Ligand binding pocket denoted by black arrow.

Next, the occupancy *n* and lifetime *τ* of the hydrogen bonds involving the four sequence differences between MpDHFR and MyDHFR in the simulations at 279K were compared. The only significant difference was at residue 103, which is Cys103 S*_γ_*…Ile99 O (forming a hydrogen bond within helix F) in MpDHFR and a Tyr103 O*_η_*…Leu78 O (forming a hydrogen bond between helix F and helix E) in MyDHFR (Figure 5). These are short lifetime hydrogen bonds, between 3 to 5 ps, and the hydrogen bond is relatively high occupancy in MpDHFR, 0.6 at 1 bar and 0.7 at 800 bar while the hydrogen bond in MyDHFR is low occupancy of 0.3 at 1 bar, but becomes higher occupancy 0.7 at 800 bar. Since residue 103 is a phenylalanine in EcDHFR and D27E EcDHFR, the side chain cannot form a hydrogen bond.

## 5. Discussion

**Figure 6:**
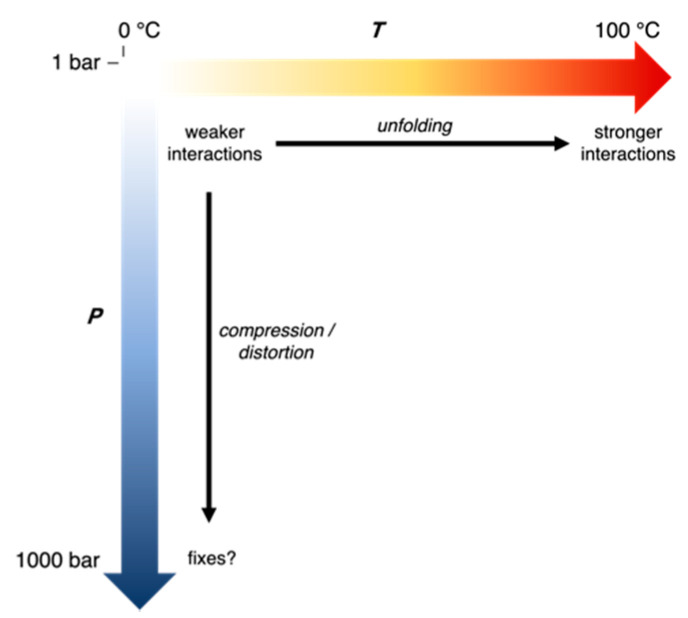
Adaptations in proteins in response to growth temperature and pressure of source organism. Black arrows indicate evolutionary stressors on proteins.

First, we present a general framework to understand adaptations in enzymes from extremophiles (Figure 6), which involves two considerations about the evolutionary history of the source organisms. The first consideration is the nature of all extremes in the growth conditions of all of the source organisms being compared, i.e., how many variables are different in their growth conditions and what the evolutionary stressors due to the conditions are. The second consideration is the direction and timescale of evolution of the organisms. Specifically, if the ancestral microbes that evolved under a different set of conditions migrate toward an extreme, only a few “staples” might appear as adaptations against the stressor while if the ancestral microbes have evolved for a long time under an extreme condition, random mutations might also optimize the “material science” properties of the protein matrix for those conditions. For instance, migrating to high temperatures might cause proteins to unfold—a strong stressor, and in the short run, the initial adaptations might be staples that are strong interactions that prevent unfolding by holding together weak parts. On the other hand, migrating to low temperatures might cause lower activity—a weaker stressor, so initially, the microbe might be able to tolerate cold just by slower growth or slight changes that improve the active site efficiency. However, for ancestral microbes that have evolved a long time under hot conditions, the protein matrix may have more interactions and thus greater compactness; while in those that have evolved a long time under cold conditions, the protein matrix may have fewer interactions and thus greater flexibility. “Staples” are easier to test by site-specific mutagenesis while the “material science” of the protein matrix might lead to better choices for proteins to bioengineer.

In this context, understanding adaptations for high pressures has been complicated because many of the comparisons have involved homologous proteins from microbes that differ in their growth pressure and temperature, and also where other aspects of their evolutionary history are not taken into account. In general, piezophilic microbes have mostly come from the deep-sea, whether from the deep-cold ocean or near hydrothermal vents. Comparisons of crystal structures of EcDHFR and MpDHFR [32] and of IMPDH from *Shewanella oneidensis* and *S. benthica* [34], both indicate larger total cavity volume in the protein from the deep-sea bacteria, which was interpreted that high pressure environments might favor more compressible proteins [34]. Although this might seem contrary to the idea that cavities promote pressure unfolding [62], it probably reflects the fact that the maximum pressures that bacteria experience in the deepest trenches is about 1.1 kbar while pressure unfolding becomes a stressor for most proteins at much higher pressures. Moreover, microbes that have evolved their “material science properties” for cold temperatures might also be better able to adapt to high pressures because they tend to have fewer interactions and are thus less compact and more compressible. However, although larger *total* cavity volume may be favored, large cavities are probably disfavored very deep in the ocean because even if complete unfolding is not a stressor, distortion might be one especially for proteins that have fewer interactions. On the other hand, microbes that have evolved their “material science properties” for hot temperatures appear to have many interactions and thus may be less sensitive to extreme pressures because they are less likely to distort or destabilize.

Of the potential adaptations for high pressure that became apparent in our simulations, Asp27Glu has been backed up by experimental data. For instance, D27E EcDHFR has increased activity with pressure as evidenced by experimental activity measurements, which was proposed to be due to the greater solvent accessibility of the active site in the mutant [36]. Our MD simulations indicate that another possible factor might be that the Thr113…Asp27 hydrogen bond in EcDHFR is a very strong “staple” and quickly becomes too strong with increasing pressure and disrupts the coupling within the protein so that loop motion is affected [44], which has been indicated experimentally as important in its enzymatic cycle [45]. Our MD simulations also indicate the Thr113…Glu27 hydrogen bond in D27E EcDHFR and two *Moritella* DHFR is a somewhat weaker “fastener” (due to greater flexibility of the glutamic acid sidechain) at 1 bar that only becomes somewhat stronger with pressure, which makes the protein behave more like EcDHFR at 1 bar [44]. In particular, the simulations show that the GH loop opens with increasing pressure in EcDHFR due to the strong coupling but does not in D27E EcDHFR. Although greater active site solvent accessibility is consistent with the greater flexibility of the Thr113…Glu 27 hydrogen bond, whether the changes in the solvent accessibility or in the coupling are responsible for the increased activity with pressure have yet to be tested. Regardless, we think this adaptation may have been for cold and, fortuitously, also makes it work better at high pressures since although all *Moritella* species so far have Glu27, they are all from cold environments but different pressure environments, from the surface to the bottom of the Mariana Trench. In addition, since DHFR from other deep-sea bacteria (including those from the bottom of the Mariana Trench [35]) have Asp 27 and not Glu27, Glu27 does not appear to be required for high pressure environments. This also implies that this mutation may not be generalizable to other proteins—for instance, Asp to Glu (nor even more flexible interactions) may not always lead to better pressure adaptation—because it may be specific to the location in DHFR. Moreover, the reason D27E EcDHFR increases with pressure while MpDHFR begins to decrease above ~500 bar (Figure 1) might because the cold-adapted MpDHFR may be beginning to destabilize since it has a *P*_u_ of ~700 bar while both wild-type and mutant EcDHFR have *P*_u_ in the range 2.5 to 2.7 kbar.

From our simulations of MpDHFR and MyDHFR, Cys103Tyr appears to be a *potential* factor in pressure adaptation in MyDHFR, although the experimental evidence is circumstantial so far. For instance, MpDHFR has *P*_u_ of ~0.7 kbar, which it should tolerate at growth conditions of *M. profunda*, which has an optimal *P*_G_ = 0.22 kbar and was isolated at a depth corresponding to ~0.28 kbar, while *M. yayanosii* has an optimal *P*_G_ = 0.8 kbar and was isolated at a depth corresponding to 1.1 kbar, suggesting that MyDHFR may need to be more robust. Of the three sequence differences between MpDHFR and MyDHFR that are unique to MyDHFR, at least some of them may be adaptations for a higher-pressure environment. Our simulations indicate that the Tyr103…Ile99 hydrogen bond between helices E and F, both in the adenosine binding domain, in MyDHFR might reduce the flexibility of the adenosine binding domain by acting as a “staple” against pressure distortion, while the intrahelix Cys103…Leu78 hydrogen bond in MpDHFR might not. This reduction of flexibility might lead to the smaller initial increase in activity with pressure seen in MyDHFR with respect to MpDHFR or D27E EcDHFR but since MyDHFR still has the Thr113…Glu27 hydrogen bond, this is better activity than wild-type DHFR. In other words, the Tyr103…Ile99 hydrogen bond may add rigidity that cancels some of the flexibility added by the Thr113…Glu27 hydrogen bond. At higher pressures, since the sequence is so similar to MpDHFR, MyDHFR should also be susceptible to destabilization in the rest of the protein at higher pressure, therefore, this is consistent with the decreasing activity with pressure (Figure 1). Thus, we might expect that Cys103Tyr mutation in MpDHFR might only have a very slightly higher *P*_u_ than wild-type and Cys103Tyr/Asp27Glu double mutation in EcDHFR might have neither increased nor decreased activity with pressure, much like the initial behavior of MyDHFR. Thus, Cys103Tyr may be a staple needed in *Moritella* DHFR because it is fragile, but might be disadvantageous in a stronger protein.

In summary, even though *Moritella* have been found at high pressures, proteins from that have evolved under mesophilic or thermophilic conditions and might be better starting points for bioengineering for piezophilicity because they might be stronger scaffolds since proteins from cold-adapted bacteria tend to be fragile. In fact, hyperthermophilic archaea may have proteins that are resistant to high pressure as well as high heat, even if they are from surface sources [26]. In addition, “fixes” for improved piezophilicity may be very enzyme specific and thus hard to identify.

## 6. Conclusions

The results here indicate that understanding the adaptations for pressure and temperature may be coupled and that evolutionary considerations may also be important. Specifically, the results suggest that replacement of Asp27 in EcDHFR by Glu27 in *Moritella* DHFR may be an adaptation for cold (or at least tolerated at cold) that fortuitously also enhances activity under pressure. The simulations indicate that the extra carbon of a glutamate increases flexibility of the Thr113…Res27 hydrogen bond while pressure increases the hydrogen bond strength and correlation of sheet F with helix B so that the net result is similar hydrogen bonding at the *P*_G_ of the microbes. In addition, the results suggest that replacement of Cys103 found in most *Moritella* DHFR by Tyr103 in MyDHFR may be an adaptation for preventing distortion of the adenosine binding domain at higher pressures that may only be advantageous for cold-adapted DHFRs, although further investigation is warranted.

## Figures and Tables

**Table 1:** Average number of hydrogen bonds and atomic fluctuations in simulations of DHFR in closed state at 279 K and different pressures; *P*_hi_ = 220 bar for EcDHFR and D27E EcDHFR and *P*_hi_ = 800 bar for MpDHFR and MyDHFR.

Protein	*P* = 1 bar		*P*_hi_	
	*N_HB_*	〈Δ*r_HA_^2^*〉 (Å^2^)	*N_HB_*	〈Δ*r_HA_^2^*〉 (Å^2^)
EcDHFR *	105 ± 2	0.57 ± 0.07	104 ± 4	0.60 ± 0.08
D27E EcDHFR *	103 ± 3	0.60 ± 0.04	109 ± 1	0.50 ± 0.04
MpDHFR	104 ± 2	0.54 ± 0.04	106 ± 3	0.53 ± 0.03
MyDHFR	107 ± 2	0.54 ± 0.05	105 ± 1	0.54 ± 0.06

* From previous work [44].

## Data Availability

The data that support the findings of this study are available from the corresponding author upon reasonable request.

## Supplementary Materials

The following are available online at https://www.mdpi.com/article/10.3390/microorganisms9081706/s1. Table S1: A more detailed Methods section is given, including the number of atoms in each simulation.
