# Supplementary material for: Adaptations for Pressure and Temperature in Dihydrofolate Reductases
Source: Microorganisms. 2021 Aug 11;9(8):1706. doi: 10.3390/microorganisms9081706 (PMC8399027; doi:10.3390/microorganisms9081706)
Supplement: Supplementary file 1 [file microorganisms-09-01706-s001.zip › microorganisms-1269836-supplementary.pdf]

SUPPLEMENTARY MATERIAL for

Adaptations for Pressure and Temperature in Dihydrofolate Reductases

Ryan W. Penhallurick, Maya D. Durnal, Alliyah Harold, and Toshiko Ichiye*

Complete Methods

A. Simulations

Information on molecular dynamics simulations programs used, as well as protein and water forcefields used can be found in Methods. The force field developed by Mackerell *et. al.*[1] was used to describe the reduced cofactor NADPH. Ligand Reader and Modeler[2] in CHARMM-GUI was used to modify the pterin ring of folate from a planar system to the partially-puckered ring of dihydrofolate (DHF), as well as to modify oxidized nicotinamide adenine dinucleotide phosphate ($NADP^+$) to the reduced form (NADPH).

Coordinates for the proteins were generated with PDB Reader;[3] specifically, termini were capped with amino and carboxyl groups, and missing hydrogen coordinates built. Starting coordinates for EcDHFR (PDB ID: 1RX2) and MpDHFR (PDB ID: 2ZZA[4]) were obtained from the PDB. For *Moritella* DHFRs, the first residue was corrected (M0V), and the C-terminal tail was built (K160) using GalaxyFill[5] in PDB Reader. For D27E EcDHFR and MyDHFR, mutations to the Ec- (D27E) and MpDHFR (C103Y, T119I, N132H, N150D) template structures were also made using GalaxyFill. Crystal waters within 2.5 Å of any modeled residue were deleted. Numbers of atoms, waters and ions for each system can be found in Table SI.

The subsequent calculations were performed in OpenMM as described briefly here; in particular, changes from default settings are noted. Initial stages of the simulations were performed using a leapfrog Verlet integrator with a time step of 0.001 ps and were maintained in the *NPT* ensemble using an Andersen thermostat[6] updated every 1000 steps and Monte Carlo (MC) barostat[7] updated every 25 steps. Each system was heated from an initial temperature of 0 K to the final temperature in 5 K intervals of 5 ps each, followed by pressurization from 1 bar to the final pressure in 20 bar intervals of 20 ps each. A harmonic restraint with a force constant of 5 kcal mol^{-1} $Å^{-2}$ was applied to the heavy atoms of the protein and ligands during heating and pressurization,[8] and then gradually decreased from 5 to 0 kcal mol^{-1} $Å^{-2}$ in 0.5 kcal mol^{-1} $Å^{-2}$ intervals for a total of 20 ps. Next, the system was equilibrated for 5 ns in the *NPT* ensemble with all harmonic restraints removed. The final stages of the simulations were performed utilizing a velocity Verlet integrator with a timestep of 0.001 ps maintained in the *NVT* ensemble using a Nosé-Hoover thermostat.[9-12] All simulations were run for an additional 100 ps and the system volumes every 1 ps were compared to that of the average volume from the last 4 ns of the *NPT* equilibration run. For all simulations at 1 bar, the closest volume less than the average volume of the *NPT* equilibration run was used to start the *NVT* production run; while for all other conditions the closest volume to the average of the *NPT* run was chosen. The system was equilibrated for another 5 ns followed by 50 ns of production run in the *NVT* ensemble.

B. Analysis

Average properties were calculated from coordinates written at 1 ps intervals except as noted. Standard deviations were calculated by block averaging over 10 ns blocks. The mean-squared fluctuations

of the protein heavy atoms $\langle \Delta r_{HA}{}^2 \rangle$ were calculated within 10 ns blocks with respect to the average structure within each block, and then averaged over all blocks.

Hydrogen bonding events were calculated in CHARMM, while MATLAB was used to calculate the average occupancies and lifetimes for each hydrogen bond pair. Two hydrogen bonds simultaneously formed with the same protein atom were calculated as two separate events. For chemically equivalent hydrogen bonding donors or acceptors of the same residue, equivalent atoms (such as $O_{\delta 1}/O_{\delta 2}$ in Asp) were combined. The occupancy, n_{ij}, was defined as the fraction of the total simulation time in which i and j are hydrogen bonded. Bifurcated hydrogen bonds were treated as a single event so that the maximum occupancy would be one. The average hydrogen bond lifetime, τ_{ij}, is the sum of the time, t_{ij}, that donor atom i is in a hydrogen bond with any acceptor atom j, over the number of hydrogen bonding events, n_{ij},

$$\tau_{ij} = \frac{1}{n_{ij}} \sum_{i=1}^{n_{ij}} t_{ij}(n) \quad (1)$$

The average overall hydrogen bond lifetime between species α and β for a simulation, $\tau_{\alpha\beta}$, is

$$\tau_{\alpha\beta} = \frac{1}{N} \sum_{i,j} \tau_{ij} \quad (2)$$

where τ_{ij} is the average hydrogen bond lifetime between any atom pair ij, respectively, and N is the total number of individual hydrogen bond pairs between the two species.

Table S1. Number of atoms in simulations.

Protein	$N_{,tot}$	N_{prot}	N_{w}	N_{K+}	N_{Cl-}
EcDHFR	43329	2489	10161	43	27
D27E EcDHFR	43332	2492	10161	43	27
MpDHFR	43515	2558	10191	40	27
MyDHFR	43456	2574	10172	41	27

References

1. Pavelites, J.J., Gao, J.L., Bash, P.A., and Mackerell, A.D., A molecular mechanics force field for NAD(+), NADH, and the pyrophosphate groups of nucleotides. *J. Comput. Chem.*, **1997**, *18*, 221-239.
2. Kim, S., Lee, J., Jo, S., Brooks, C.L., Lee, H.S., and Im, W., CHARMM-GUI ligand reader and modeler for CHARMM force field generation of small molecules. *J. Comput. Chem.*, **2017**, *38*, 1879-1886.
3. Jo, S.W., Cheng, X., Islam, S.M., Huang, L., Rui, H., Zhu, A., Lee, H.S., Qi, Y.F., Han, W., Vanommeslaeghe, K., MacKerell, A.D., Roux, B., and Im, W., CHARMM-GUI PDB Manipulator for Advanced Modeling and Simulations of Proteins Containing Nonstandard Residues, in *Biomolecular Modelling and Simulations*, T. KarabenchevaChristova, Editor. 2014, Elsevier Academic Press Inc: San Diego. p. 235-265.
4. Ohmae, E., Murakami, C., Tate, S.-i., Gekko, K., Hata, K., Akasaka, K., and Kato, C., Pressure dependence of activity and stability of dihydrofolate reductases of the deep-sea bacterium *Moritella profunda* and *Escherichia coli. Biochim. Biophys. Acta,* **2012**, *1824*, 511-512.
5. Coutsias, E.A., Seok, C., Jacobson, M.P., and Dill, K.A., A kinematic view of loop closure. *J. Comput. Chem.*, **2004**, *25*, 510-528.
6. Andersen, H.C., Molecular-dynamics simulations at constant pressure and/or temperature. *J. Chem. Phys.*, **1980**, *72*, 2384-2393.
7. Aqvist, J., Wennerstrom, P., Nervall, M., Bjelic, S., and Brandsdal, B.O., Molecular dynamics simulations of water and biomolecules wit a Monte Carlo constant pressure algorithm. *Chem. Phys. Lett.*, **2004**, *384*, 288-294.

8. Sheinerman, F.B. and Brooks, C.L., A molecular dynamics simulation study of segment B1 of protein G. *Protein Struct. Funct. Genet.*, **1997**, *29*, 193-202.
9. Nosé, S., A unified formulation of the constant temperature molecular dynamics methods. *J. Chem. Phys.*, **1984**, *81*, 511-519.
10. Hoover, W.G., Canonical dynamics: Equilibrium phase-space distributions. *Phys. Rev. A,* **1985**, *31*, 1695-1697.
11. Martyna, G.J., Klein, M.L., and Tuckerman, M., Nose-Hoover Chains - The Canonical Ensemble via Continuous Dynamics. *J. Chem. Phys.*, **1992**, *97*, 2635-2643.
12. Martyna, G.J., Tuckerman, M.E., Tobias, D.J., and Klein, M.L., Explicit reversible integrators for extended systems dynamics. *Mol. Phys.*, **1996**, *87*, 1117-1157.
